# Bringing the social into vaccination research: Community-led ethnography and trust-building in immunization programs in Sierra Leone

**DOI:** 10.1371/journal.pone.0258252

**Published:** 2021-10-22

**Authors:** Luisa Enria, Joseph S. Bangura, Hassan M. Kanu, Joseph A. Kalokoh, Alie D. Timbo, Mohamed Kamara, Maligie Fofanah, Alhassan N. Kamara, Adikalie I. Kamara, Morlai M. Kamara, Ibrahim Sorie Suma, Osman M. Kamara, Alusine M. Kamara, Alhajie O. Kamara, Abu B. Kamara, Emmah Kamara, Shelley Lees, Mark Marchant, Mariama Murray

**Affiliations:** 1 Department of Global Health and Development, London School of Hygiene & Tropical Medicine, London, United Kingdom; 2 Kambia District Health Management Team, Ministry of Health and Sanitation, Government of Sierra Leone, Kambia, Sierra Leone; 3 Kambia District Community Health Workers Programme, Kambia, Sierra Leone; 4 National AIDS Control Programme, Ministry of Health and Sanitation, Government of Sierra Leone, Kambia, Sierra Leone; University of Toronto, CANADA

## Abstract

**Background:**

Vaccine hesitancy is a complex, contested social phenomenon and existing research highlights the multifaceted role of trust in strengthening vaccine confidence. However, understanding public engagement with vaccination through the lens of (mis)trust requires more contextual evidence on trust’s qualitative determinants. This includes expanding the geographic focus beyond current studies’ focus on High Income Countries. Furthermore, obstacles remain in effectively integrating social science findings in the design of vaccine deployment strategies, and in ensuring that those who implement interventions and are affected by them are directly involved in producing knowledge about vaccination challenges.

**Methods:**

We piloted a community-led ethnographic approach, training Community Health Workers (CHWs) in Kambia District, Sierra Leone, in qualitative social science methods. Methods included participant observation, participatory power mapping and rumour tracking, focus group discussions and key stakeholder interviews. CHWs, with the support of public health officials and professional social scientists, conducted research on vaccination challenges, analysed data, tested new community engagement strategies based on their findings and elicited local perspectives on these approaches.

**Results:**

Our findings on vaccine confidence in five border communities highlighted three key themes: the impact of prior experiences with the health system on (mis)trust; relevance of livelihood strategies and power dynamics for vaccine uptake and access; and the contextual nature of knowledge around vaccines. Across these themes, we show how expressions of trust centered on social proximity, reliability and respect and the role of structural issues affecting both vaccine access and confidence. The pilot also highlighted the value and practical challenges to meaningfully co-designed research.

**Conclusion:**

There is scope for broader application of a community-led ethnographic approach will help redesign programming that is responsive to local knowledge and experience. Involving communities and low-cadre service providers in generating knowledge and solutions can strengthen relationships and sustain dialogue to bolster vaccine confidence.

## Background

The COVID-19 pandemic has brought vaccination to the fore of global public debate. The media and policymakers have presented the advent of a licensed COVID-19 vaccine as a silver bullet to end the crisis. Alongside discussions about inequities in access to vaccines, efforts to ensure sufficient vaccination coverage have placed emphasis on countering misinformation and potential vaccine ‘hesitancy’ amongst certain sections of the population [1–3]. Defined as “refusal, delay or acceptance with doubt about vaccine usefulness” [3], vaccine hesitancy is however not a new concern. In 2019, the World Health Organisation (WHO) listed it amongst the top ten threats to global health, and member countries have been encouraged to “develop a strategy to increase acceptance and demand for vaccination” [4, 5]. Measles outbreaks in Europe and the United States have been linked to declining vaccination coverage and a recent comparative study shows declining confidence rates across several countries between 2015–19 [6].

Public perceptions of vaccine hesitancy have commonly associated it with “knowledge deficits”, a frame that posits hesitant individuals and groups as having insufficient understanding of the value of vaccination [7]. In this “oppositional framing of the problem as a conflict of science versus ignorance” [7], hesitancy is reduced to misconception or attributed to unscientific beliefs. The “knowledge deficit presumption” has been vigorously challenged, and more critical approaches to understanding experiences and perspectives of vaccination have emerged. These approaches recognise the complexity of the phenomenon, understanding vaccine confidence and hesitancy through a range of sentiments and social dynamics. A summary of the factors influencing attitudes to vaccination for example refers to the 5Cs: confidence, complacency, convenience, risk calculation and collective responsibility [8]. In these discussions, trust is identified as being essential, including trust in the product (i.e., the vaccine), healthcare providers, policymakers and information, as well as trust in society and historical influences [9]. A focus on trust allows us, as Goldenberg suggests, to reinterpret vaccine hesitancy from a knowledge deficit to “a problem of mistrust of scientific experts and institutions” [7]. However, whilst most agree that trust is important for understanding vaccine hesitancy, Larson et al note that research so far has only offered a partial picture of its drivers [9]. To some extent this is because trust, despite being ubiquitous in social scientific analysis, has remained a slippery concept. Broadly, understood as a “social orientation towards the future”, an affective relation or calculation of risk, efforts to capture the nature and drivers of trust have been critiqued as “ill-suited to unpack the complex, manifold ways in which trust is conceptualised, formed and lived around the world”, and “too narrowly focused on Western contexts” [10].

Understanding public engagement with vaccination through the lens of (mis)trust, therefore, requires careful contextual analysis of a range social experiences and perspectives. This agenda also necessitates expanding the geographic focus of existing evidence, as the vaccine hesitancy literature has focused disproportionately on High Income Countries (HICs) [8]. Inspiration for bringing the social into vaccine research can be found in anthropological work on local engagements with scientific research, including vaccine trials, in low-income settings. These studies have situated (mis)trust in specific histories, including of colonialism and extraction, and political subjectivities, as well as exploring how engagement with scientific technologies such as vaccines can generate new identities, relations and socialities [11–17]. In the realm of vaccine hesitancy, Obadare’s analysis of the 2003 polio controversy in Northern Nigeria similarly explains contentions by paying attention to local and global political-religious contexts, to argue that “the crisis is best seen as emanating from a dearth of trust in social intercourse between ordinary citizens and the Nigerian state on the one hand, and between the same citizens and international health agencies and pharmaceutical companies on the other” [18, p265]. From these perspectives, we can challenge hesitancy frameworks that focus on individual responsibility, looking instead to how social, political, and economic factors affect vaccine acceptance, anxieties and refusal. Situating concerns and hopes around vaccination in this broader social analysis then also allows us to engage with the interplay between structural realities and individual decisions, including taking into account the ‘access supply side’ alongside demand for vaccines [8].

The recognition of a need for contextual, in-depth understanding of the social dimensions of global health challenges has undoubtedly grown in recent years. Perspectives from the social sciences are increasingly acknowledged as crucial to ensure interventions are tailored to the setting where they are implemented, taking into account the interaction of different social factors determining engagement with biomedical interventions [19–22]. In the field of epidemic response, for example, anthropological contributions have critiqued risk communication and behaviour change models that envision ‘culture’ as a barrier, proposing instead that response measures take local knowledge, priorities and experiences as a starting point [23, 24].

Despite these efforts, challenges remain. The recognition that social dimensions are important has not always translated into systematic approaches for effectively operationalizing social science findings, for example in terms of how to integrate them into community engagement campaigns around immunization. Partly, this is because of the way that existing structures of research work, whereby affected communities, community workers and public health officials working on the ground during these campaigns are rarely directly involved in the process of generating knowledge about and devising solutions for the local determinants of vaccine hesitancy. Knowledge production in global health has come under scrutiny in debates about decolonising and democratising research [25, 26]. Widening the range of voices and perspectives included in the evidence base driving global health interventions is crucial for both justice and effectiveness reasons. As for other health challenges, efforts to develop interventions that strengthen vaccination confidence and coverage can only be sustainable if they are led by local knowledge, responsive to the experience of affected individuals and communities and directly shaped by those closely involved in the delivery of vaccines on the ground.

In this paper, we present findings from an exploratory project aiming to integrate community-led ethnographic research into the development and implementation of strategies to address vaccine hesitancy and other immunisation challenges in Sierra Leone at the community level. The project trialled a social science training for Community Health Workers (CHWs), who then conducted participatory, ethnographic research on vaccination challenges in five communities and worked with these communities and district public health officials to translate these findings into a new engagement strategy. CHWs then tested key aspects of this strategy and consulted community members on the appropriateness of the recommendations. The paper contributes original findings on the social dimensions of vaccination challenges in rural Sierra Leone and offers reflections on the process of conducting and operationalising community-led qualitative research to support localised solutions for strengthening vaccine confidence and immunisation coverage.

The pilot project was conducted in Sierra Leone’s Kambia District between September 2019 and March 2020. It was implemented through a partnership between the London School of Hygiene & Tropical Medicine, University of Bath and the Kambia District Health Management Team, the local representation of the Ministry of Health and Sanitation.

The paper is divided into four sections. Firstly, we introduce the project’s context and its inception in long-term discussions and partnership between social scientists and public health officials in the District, originating in shared experiences during the response to Sierra Leone’s Ebola outbreak. Secondly, we outline the research methodology and the design of a process for translating findings into operational recommendations. Thirdly, we present key findings from a first round of research in five border communities and reflecting on a new community engagement strategy based on research findings. In the Discussion we consider broader applications for this approach and potential limitations.

### Kambia’s community health workers’ social science project

Kambia District lies on Sierra Leone’s North-Western border with Guinea with 384,932 inhabitants across ten chiefdoms, served by one hospital and 70 other health facilities including Community Health Centres (CHCs) and Peripheral Health Units (PHUs). The District has ranked lower than other parts of the country in terms of vaccination coverage, reporting under 70% on most vaccinations and 48% for the second MMR dose in 2019 (though a marked improvement from 19% in 2017). Concerns around vaccination coverage were raised especially in January 2019, when Kambia became one of two Districts to be affected by a measles outbreak. Like the rest of the country, Kambia was also adversely hit by the 2014–16 Ebola outbreak, which had significant repercussions on the health system, depleting physical and human resources. During this time, the District also became the site of an Ebola vaccine (EBOVAC) trial [12, 27–29].

Collective research design and implementation were at the heart of this project and warrant a detailed description, especially to generate insights for future uses of this community-led ethnographic approach. Building and maintaining a meaningful partnership over time was a key component. The EBOVAC trials had a social science component, led by SL and LE, focused on researching local experiences of the Ebola epidemic and perspectives on biomedical research. Through this work, and through a subsequent project on the Anthropology of emergency Vaccine Deployment (AViD), a long-term partnership was established with the District Health Management Team (DHMT), the representation of the Ministry of Health and Sanitation at District level. This partnership, and initial research findings from the AViD project on the barriers and facilitators of vaccine deployment in the District, ignited a conversation about the importance of identifying reasons behind Kambia’s relative lower rates of vaccination and possible solutions. Received wisdom in the district had put this down to a broadly defined problem of ‘refusal’ and assumptions about a lack of information about vaccines. These explanations risked obscuring significant ‘supply side’ issues and did not identify the drivers of vaccine hesitancy. DHMT colleagues reported experiencing significant challenges during immunisation efforts in specific parts of the district, particularly around the border, where they encountered lower uptake and, in some cases, active refusal. However, they also noted that they did not have any concrete evidence as to why this might be the case. This meant that community engagement campaigns around vaccines had often relied on ready-made messaging, produced at national level and standardised across districts. DHMT colleagues felt that this was failing to directly address the specific issues that may be determining hesitancy in different communities. This became particularly visible in January 2019 as the DHMT had to respond to a measles outbreak in these border areas.

Following these conversations, LE and DHMT leads convened as a team and drew up a plan for transferring some lessons learned in our previous projects on how to integrate social science findings in clinical and epidemic response [28, 30]. The plan was to develop a research process to better understand experiences and perspectives of vaccination to support the DHMT’s programming for routine and emergency vaccination. Our initial team was made up of LE as the lead social scientist, the District Medical Officer (MM then JSB), the DHMT Social Mobilisation Manager (HK) and Extended Programme on Immunization (EPI) Manager (JK), and two social science researchers (ADT and MK) based at the DHMT and trained in qualitative data collection and analysis. We decided to take lessons from our previous work further and consider avenues for developing a community-led approach. In this case this meant involving Community Health Workers (CHWs) in the research process and in operationalising the findings into strategies that were responsive to their communities’ needs. Across different contexts, CHWs have been shown to make important contributions to health service delivery, such as immunisation, including by improving reach, uptake and quality of services, although their contribution is not always socially recognised [31, 32]. CHWs were introduced in the Sierra Leonean health system in 2012, with a focus on their role in health education and Integrated Community Case Management. They have been considered an important addition to a long-term process of decentralising health services, addressing the challenge of limited human resources in healthcare and against the backdrop of the Free Healthcare Initiative set up by the Government of Sierra Leone in 2010 to increase health coverage across the country, with an emphasis on efforts to reduce the country’s high levels of maternal, new born and child deaths [33]. Although the implementation of the National CHW Policy was interrupted by the 2014 Ebola outbreak, CHWs were active in supporting the epidemic response, taking a key role in community engagement, detection and contact tracing amongst other activities [34]. In 2017, CHWs were trained and started operating in selected Districts, including Kambia. The National CHW policy for Sierra Leone refers to the WHO’s definition, suggesting that CHWs “should be members of the communities where they work, should be selected by the communities, should be answerable to the communities for their activities, should be supported by the health system but not necessarily a part of its organization, and have shorter training than professional workers” [33, p4]. CHWs in Sierra Leone are supposed to receive some remuneration, though this has been found to be often delayed and insufficient at around $18–24 per month [35].

As the closest health workers to community members, CHWs are expected to improve access to healthcare, and be involved in all areas of community health, from nutrition, water and sanitation interventions to community surveillance and referrals. Importantly, CHWs are also engaged in social mobilisation and health education around vaccinations. Their “interface role between communities and health systems” has been found to be “critical because of their embedded positionality and the trusting relationships they (often) have” [35, p. 12]. This “interface role” and “embedded positionality” made them also particularly well suited to act as community researchers, with an ideal vantage point to explore citizens’ experiences and perspectives of vaccination.

## Methodology

In August 2019, members of the DHMT community engagement and EPI teams drew up a list of five border towns or villages that had lower coverage levels or where the engagement team had experienced what they perceived to be community avoidance or refusal during immunisation drives. Ten CHWs associated with the relevant Peripheral Health Units (PHU) were invited to a one-week training in September. Three of these CHWs were ‘peer supervisors’, that is CHWs with additional training in supporting others in conducting their role, while the other seven were regular CHWs. All were from the border chiefdoms where they five communities were situated and as such were seen as ‘sons of the soil’, however they were not necessarily living in the specific village where they conducted the research.

LE developed a tailored, intensive social science training programme aimed at a lay audience, framed as an introduction to qualitative research with an emphasis of ethnographic perspectives and participatory methods. It included both theoretical and practical sessions on topics including foundational concepts in qualitative social science, the interpretivist paradigm, participant observation and writing ethnographic notes, conducting in-depth interviews and focus group discussions and participatory activities including power mapping and rumour tracking group exercises. The training workshop was led by our experienced social scientists and the DHMT supervisory team; it was developed to match the CHWs’ experience. The training was not intended to be exhaustive, as the bulk of the learning was done through practice with close daily support from our social science team. The workshop was also an opportunity define a broad research agenda to explore experiences and perspectives of vaccination, studying hesitancy contextually and situating it in a broader analysis of vaccine deployment and uptake challenges in the borderlands. This agenda was then to be refined iteratively through the process of research.

A key message in the training workshop was to encourage CHWs to take this opportunity to act as “strangers” in communities they knew well, observing aspects of their villages and towns they may have not paid attention to before and allowing themselves to be surprised by some of the findings from the research. As one of the guest presenters at the workshop put it: researchers should arrive in the field, even if they know it well, with an “empty cup” to be filled with new knowledge. With this in mind, we began the workshop by listing and ranking the assumptions that training participants made about why there may be challenges in achieving high vaccination coverage rates in their communities, to be contrasted with our findings at the end of the project.

After the training, in October 2019, the CHWs began their research by holding meetings in their five communities, inviting members of the DHMT to support them to introduce the aims of the research. The CHWs then worked in pairs, visiting the same community every day. Over the course of a month, they each wrote daily ethnographic diaries on observations on their social interactions in the community, describing livelihood strategies, social relations across the border and movement patterns, community members’ relationships with health workers and their experience of health services, perceptions of illness and disease, opinions about and experiences of routine and emergency vaccination, memories of Ebola and other outbreaks, amongst other topics. CHWs were also asked to write reflections of their own experience as researchers and how, if at all, it affected their understanding and conduct of their role in these communities. They also conducted two key informant interviews with community authorities (Total = 10) and two focus group discussions in each community (Total = 10), with a maximum of 10 participants each. The FGDs included one power mapping workshop, where participants were asked to map patterns of formal and informal influence, sources of influence (e.g., traditional roles, wealth or knowledge) and to identify “change makers” in facilitating dialogue around vaccines. The other FGD was on vaccine confidence more directly, aiming to stimulate discussions amongst participants about experiences of vaccination, community engagement during immunisation campaigns and to elicit recommendations for better strategies. Participants for key informant interviews and FGDs were identified through initial community consultations and to ensure representation of key social characteristics, including gender and occupation. CHWs received close support from two trained social science researchers (ADT and MK) based at the DHMT, who also conducted regular refresher and troubleshooting sessions. These researchers then translated from Themne into English and transcribed the data, including verbatim hand-written daily field diaries from all CHWs, interview and FGD audios, in November 2019. For two FGDs and one interview, summary notes of audios were written by one CHW with the support of a data analyst as they were conducted in Soso.

In December 2019, we came back together as a team for a collaborative data analysis workshop, with the CHWs and the DHMT facilitated by the lead social scientist and the DHMT data analysts. This started with unstructured discussions about findings, moving gradually towards a more systematic approach to developing themes through participatory activities and close readings of transcripts and observation notes. One session focused entirely on the experience of CHWs conducting the research, engaging them in a reflexive exercise about how their assumptions about the vaccine hesitancy challenge might have changed and, more generally, how being involved in in depth qualitative research had affected their views of themselves and their community. The second half of the workshop considered how each theme from our collective analysis could be translated into concrete recommendations for improving community engagement around vaccination, including developing new messages and activities, discussed in more detail below Whilst the focus was on community engagement, recommendations also included suggestions on vaccine delivery (the ‘supply side’) for the DHMT to take into consideration.

In January 2020, the CHWs went back to their communities to implement some of the recommendations they had put forward, focusing in particular on new community engagement and communication strategies to improve vaccine confidence. They kept regular notes of their observations as they conducted these activities. In February and March 2020, the CHWs conducted another round of qualitative research to assess community perceptions of the new activities and messages they had trialled the previous month. This included seven interviews (four with community members and three with health workers) and five FGDs in total, as well as continued daily notes from participant observation in communities and health centres. Table 1 summarises the research process and timeline.

**Table 1:** Research process and timeline.

Research Process	Timeline
Step 1: CHW Recruitment	August 2019
Step 3: One-week intensive training	September 2019
Step 4: CHW conduct one month of qualitative research on experiences and perspectives of vaccination	October 2019
Step 5: Collective Data analysis and design of new strategy	December 2019
Step 6: Implementation of new strategy	February 2020
Step 7: CHW conduct one month of qualitative research to gather perspectives on new strategy	March 2020

The project received ethics approval from the University of Bath Social Science Research Ethics Committee Ref S19-071 (where LE was employed at the time of research) and from the Sierra Leone Ethics and Scientific Review Committee. Informed consent was obtained in writing for interviews and Focus Group Discussions, transcripts were anonymised, and names of individuals in ethnographic field notes were anonymised at the point of transcription.

## Findings

### Key themes

The CHWs’ research generated a wide range of empirical findings around experiences and perspectives of vaccination in Kambia’s border communities. These can be summarised in three broad thematic areas: experiences of health services; community dynamics and knowledge around vaccination.

The team’s ethnographic approach made it possible to see how narratives of (mis)trust around vaccination were couched in broader reflections on the health system and particularly past interactions with healthcare workers. Interlocutors for example expressed their concern to CHW-researchers that seeking care at a government health facility may require paying money that they did not have, including both payment for drugs and services and high transport cost for travel on poor roads in rural chiefdoms. These concerns were mentioned to explain reluctance to visit health centres and decisions to opt instead for alternative pathways such as private pharmacies or traditional healers and traditional birth attendants (TBAs) that were in some cases viewed as more affordable and accessible. Indeed, in their discussions, CHWs recorded their fellow residents’ experiences of having to pay for vaccines or vaccination cards, despite the fact that under Sierra Leone’s Free Healthcare Initiative, (FHCI) children under five should not pay for these services. At the same time, poor communication around the parameters of the FHCI meant that being asked to pay for a service that fell under the cost recovery scheme (i.e., services not eligible for free healthcare) was perceived as a form of corruption.

Listening to communities’ concerns, whilst also being aware of working conditions in the health-centres, CHWs were able to spot these tensions and communication gaps between healthcare workers and their communities. An example of this was the challenge of frequent drug stockouts which led to health staff having to ask patients to buy their own, or the fact that nurses were volunteers and at times had to supplement their income by ‘selling’ vaccination cards. These structural issues were exacerbated these experiences of poor service, contributing to patients’ mistrust.

Closely related to anxieties around the financial burden of formal healthcare, the research highlighted a shared feeling of humiliation experienced by patients at rural health centres. As one CHW recorded in his notes:

“*One man raised a concern by saying that he sent his wife to the health centre for his child to have the marklate [vaccine]*. *When she arrived at the health centre*, *the nurse told his wife*: *‘Look how dirty you have come to the centre’*. *His wife almost wanted to cry because she is even older than the nurse*, *and there were many people at that time in the health centre*”.

Patients felt that they were looked down upon and discriminated against based on their appearance or lack of education. Similarly, in these border communities, there was a widespread perception that Guinean patients, despite not being within Kambia’s catchment area, were treated better and quicker because they could afford higher fees.

These experiences set the foundations for a generalised sense of mistrust in the health system that had significant implications for vaccine uptake. This was based both on past interactions but also on fears borne out of assumptions about what might happen if one were to visit the health centre, which led to pre-emptive avoidance. In their discussions, feelings of mistrust in the healthcare system and healthcare workers were directly connected to explanations for avoidance of routine immunisation and vaccination campaigns. One CHW for example recounted a woman’s explanation of why when vaccinators came to their village some parents would hide their children:

*“[She] said one of her sisters was pregnant*, *and she was about to deliver*, *she…had no access to an ambulance or a motorbike to take her to the health centre*. *So*, *she tried the Traditional birth Attendant (TBA) who delivered*, *and it went successfully*. *The next day*, *she visited the health centre*, *and she was asked to pay a fine for fifty-thousand leones* [approx. USD 5]*…*.*With all the challenges and problems*, *she said that is why when they see health workers in their community they normally run to the bush and make the children go inside”*

It is interesting to note here that the fines for home deliveries that were introduced to address high maternal mortality rates were having a counter-productive effect. Rather than attending to the reasons why people may deliver at home or even prefer seeking help from a TBA, the financial disincentives were exacerbating mistrust, making women feel not only that their challenges were not being acknowledged but that they were actively being punished. Following these narratives, it was also possible to start identifying, conversely, some of the determinants of trust in this context. Comparisons were often made between health staff and alternative providers, such as traditional healers (‘herbalists’) who some saw as more trustworthy:

“*I decided to visit the herbalist because he can have my drugs for treatment in an easy or normal way*. *When you visit the herbalist*, *straight he will go to the bush and have my medicine… the delay in the PHU leads to death*. *The herbalists*, *they also [provide the service] and you pay later*, *some don’t receive money until you are better*, *while in the PHU you can’t have access like that*. *[*…*] If it’s an illness that will lead to death*, *the person will die for no good reason*. *So*, *for me*, *I prefer to go to the herbalist*”

The relative trustworthiness of herbalists and TBAs was deliberated in terms of their embeddedness in community structures. Social proximity, denoted for example by the use of terms such as “uncle”, highlighted this. Healers were also trusted as the first port of call to determine whether an illness was a ‘hospital sick’ or a ‘country sick’ (e.g., caused by witchcraft) to help them decide whether they should go to a health centre. A thorough discussion of local understandings of illness and disease is beyond the scope of this paper. However, it is important to note that in this and other research across the District, this distinction was frequently cited to explain health-seeking decisions. Alongside affordability and social proximity, healers were expected to determine whether an illness should (or could) be treated in hospital or whether it required traditional remedies because it had been caused by witchcraft, a ‘country sick’. If the illness was found to be a ‘country sick’, respondents argued, visiting a health centre that practiced Western medicine would only worsen the situation.

These characteristics did not denote a categorical preference for traditional healers or TBAs; individual healthcare workers could also achieve trustworthiness according to these characteristics. Emphasis was placed in particular on expressions of respect when respondents praised individual nurses, citing for example instances when they would help mothers read vaccination cards and translate appointments into the Islamic calendar. These reflections were illustrative of the possibilities for building trust through interpersonal relations. In their own reflections whilst carrying out the research, the CHWs similarly noted opportunities for building trust in their own work. They noticed that in their new role as researchers they were spending more time in their communities and “*mixing well*” with people in the villages, and as a consequence they were able to have more frank discussions.

Rather than focusing solely on vaccination encounters, these considerations highlighted the wide-reaching effects of mistrust, as these experiences were used to contextualise parents’ reluctance to engage with the formal health system, including for immunisation. In this first thematic area, therefore, the CHWs’ research offered some preliminary insights into what might drive trust in the particular context of their communities. They support Broch-Due and Ystanes’ intimation that rather than focusing on trust “as a thing” we consider the act of trusting, and in particular how it “realizes itself in the intersubjective space *between* persons” [10, p54]. In the particular interactions recorded by the CHWs, trustworthiness can be summarised by referring to three characteristics: social proximity, reliability and respect. Lack of respect was experienced as judgement and discrimination based on appearance, education or social position and contrasted for example with some herbalists’ social embeddedness and appreciation of the financial struggles of rural households. Communication gaps meant that what may be understood to be larger challenges in the functioning of the health system, were interpreted as dishonesty or lack of reliability, undermining trust in individual health workers.

The second theme highlighted the significance of community characteristics, including livelihoods and power dynamics, for an understanding of vaccine uptake. The five communities where the work took place lie on the border between Sierra Leone and Guinea. Borderlands tended to be seen by national and public health officials as ‘problematic’ because frequent circular movement, including through informal border crossings, was seen to facilitate the spread of infectious diseases and to undermine effective vaccination coverage [36]. CHWs’ community profiling offered more nuanced descriptions of cross-border relations and movement patterns, highlighting mismatches between borderland livelihoods and the organisation of immunisation campaigns. For example, the research highlighted that travel to trade in the nearby Guinean town of Pamlap meant that parents often had to miss dates for vaccination. This was particularly the case for traders of perishable goods who simply could not delay their sales to fit around the vaccination campaigns. Similarly, scarcity of land on the Sierra Leonean side had pushed people to farm and hunt across the border. Close linguistic and cultural ties, marriages and strong social networks across the border meant that frequent border movement was to be expected, especially at particular times of the day and year—e.g. around the time of harvest, celebration and trading hours. In addition, CHW’s conversations with both community members and health staff brought up concerns around the fact that Guineans living near the border often preferred to access healthcare in Sierra Leone, including for vaccinations, resulting in potential miscalculations in the catchment area populations for vaccine coverage.

Through their participatory power mapping exercises and their ethnographic observations, CHWs’ were also struck by the complexity and diversity of power dynamics across different communities. Reflecting on how previous social mobilisation campaigns had taken a standardised approach, engaging similar types of stakeholders, the power mapping workshops made clear that in each community, different kinds of people had the power to influence opinions and behaviours around vaccination. Having a formal position did not mean that the individual was trusted. Conversely, some of those with most sway over public opinion had no position at all and as such were missed out during mobilisation campaigns. In some communities for example, chiefs, who tend to be the gatekeepers of public health and development activities [37], were in charge, whilst in others they deferred to other individuals who had more influence, whether because of their knowledge, financial means or social recognition. Previously undervalued groups, such as *attaya* bases (coffee shops) and social clubs were particularly important for bringing people together, whilst political actors such as local Councillors were less trusted because they were associated with the partisan nature of politics and often absent. Power was also important to consider at household level, as CHWs observed that whilst women often took care of their children’s health and in some cases wanted to bring them to the clinic to be vaccinated, they were not always able to make final household decisions.

Communities’ recent histories also mattered, in particular in relation to health interventions. Like the rest of Sierra Leone, memories of the 2014–16 Ebola outbreak remained vivid in these five border communities. This included recounting experiences with local leadership during the epidemic as in some villages, chiefs and other authorities ensured that their people complied to by-laws. Memories of Ebola were also invoked in comparisons with immunisation drives, as people criticised public health efforts led by “strangers” from outside the community. CHWs were concerned about the possible implications of even bringing up the question of the effects of Ebola on contemporary experiences, as they worried this would create suspicion, given the lingering concerns and mistrust surrounding an outbreak response that had felt often external, disruptive and even violent in marginalised communities. They noted that at the beginning of the research process, their questions and observations in villages raised concerns that it may be foreshadowing the arrival of a new disease.

A third set of findings had to do with different perspectives on knowledge around vaccination. At the start of the project, trainees stated their assumptions about the topic of research. Prominent amongst these was the notion that hesitancy was due to a “lack of awareness”—replicating the “knowledge deficit” model discussed above. This was associated particularly with rumours around vaccination. Undoubtedly, our research identified a number of rumours, including some reflecting anxieties about possible associations between vaccination and infectious disease outbreak. Recent experiences with Ebola came up again, for example again respondent put it: “*some associate the vaccine with Ebola—[they say] that the vaccine will bring Ebola back*, *that is the secret*!” Similarly, the CHWs’ research during the initial days of the COVID-19 pandemic in March 2020, showed that concerns, such as the fear that health-workers may inject patients with the disease, were cited to explain avoidance of health centres and missed immunisation appointments.

However, taking all the findings from this research together highlighted that engagements with vaccination were more complex. The dominance of generalised mistrust in the findings for example allowed us to situate these rumours in broader anxieties associated with health workers and interventions [24, 38]. In addition and contrary to their initial expectations, the CHWs’ research showed that most people they interacted with actually found vaccines to be important and valued. Vaccine-specific concerns were primarily linked to fears of side-effects as parents said they were concerned that their children might be unwell after taking vaccinations. These fears were not simply about a reasonable concern with children’s wellbeing; they were also tied to broader concerns about the implications of having to take a member of the family to the health centre because of the experiences and mistrust noted above. Although recent experiences of outbreaks evoked painful memories and in some cases mistrust of health staff, they had also made communities more aware of the challenges associated with epidemic diseases. These insights undermined assumptions, shared both by CHWs and district public health officials, that rumours and concerns or avoidance of health centres were caused primarily by a lack of knowledge. In contrast, their findings showed the importance of considering other challenges for uptake, including those that did not reflect vaccine hesitancy per se, but rather more contextual or structural issues, such as missed visits due to livelihoods across the border or concerns about finances or discrimination associated with accessing healthcare.

### CHWs’ experiences

One of the most significant lessons from this project was the CHWs’ reflexive engagement with the research process and what they perceived to be its impact on their work, identity and perspective on vaccination challenges.

The CHWs reflected on how through the project they developed research and writing skills and argued that the process of conducting research had brought them closer to the community where they worked, facilitating different kinds of interactions. One CHW for example told the group that whilst he knew the community where he worked very well and lived close by, the relationship he had with them was changed by the nature of participant observation and that this had helped him build trust with residents. Being in the village regularly, sitting with community members, and participating in their daily activities had meant that: “*Now if there is any information in the community*, *they call me*, *they’ll say*: *Man come here*, *we have something to tell you*. *In the past they wouldn’t have even called me*.” Another colleague agreed and recalled how simple activities such as pounding rice and cooking food with the chief’s family, meant that he was able to conduct better research and gain long-term trust for future community engagement: “*due to this research*, *now they are used to me*, *so if I give them any information now*, *they will take it to be important*”.

The CHWs also felt that the research process had encouraged them to change the ways they did their work:

“*It has even changed my work as CHW*. *How*? *Because normally as a CHW*, *if the vaccine comes*, *I would just go to the chief and say*: *“Chief*, *the marklate has come*, *o*! *Let’s go tell our people”*. *But this research has told me about the people who I should really meet in the community*, *because they have a say there*, *not just the chief all the time*! *Maybe that man who sits in the corner*, *who doesn’t show up and you might not know he has influence over vaccination*, *he will be able to help us*. *I also learned that we need to involve people in the communities in the ‘marklate business’* [vaccination], *it shouldn’t just be us in the hospitals*.”

Similarly, for this CHW the research brought on a difficult reckoning, as it caused him to observe from a fresh perspective the humiliation experienced by some community members when they visited the health clinics:

“*This research it changed me*! *I got the experience that you can offend someone in a way you don’t even know*…*One day I went to go to the hospital, the way I saw how the nurses treat our people, I wrote it down and, in the evening, I looked at the paper and said: so, this is how we offend people!*”

In addition to suggesting new ways of working, the research also generated new understandings of what vaccination challenges were in the first place, moving beyond explanations focused on ‘negligence’ or ‘lack of understanding’ to grapple with the complex of factors from mistrust in the health sector to cross-border livelihoods leading to higher defaulter rates.

Of course, CHWs also encountered challenges in the research process, ranging from logistical issues in trying to reach their communities, or difficulties in arranging FGDs to the subtler tensions between their role as researchers and their training in health education and community engagement. Separating the two roles was not only difficult for the CHWs, who found themselves fighting the instinct to immediately ‘sensitise’ before listening to communities’ concerns, but also for their respondents who saw them inevitably as representatives of the health facility. To some extent this reflected the tension inherent in being a CHW, having to represent both the interest of their fellow residents and those of public health officials, but the research added a new layer of complexity to this balancing act. In some instances, the CHWs reported being called ‘journalists’ by their communities, expressing concerns that they were there to ‘gossip’. The process of building trust through daily encounters and participation was key in encouraging respondents to open up to the CHWs.

### Integrating social science findings into a new District strategy on vaccine confidence

In our December 2019 collaborative data analysis workshop, the team considered how some of these findings could be translated into actionable changes to the District’s strategy for improving vaccination coverage and addressing vaccine hesitancy. Some recommendations were put to the DHMT to integrate into their long-term planning and for future vaccination campaigns. These included for example suggestions that prior to a campaign, public health officials conduct power mapping activities to ensure the right leaders are involved in community engagement efforts. The EPI and community engagement team were also encouraged to consider the timing of campaigns and outreach (both time of the day and time of the year) around the farming cycle and trading commitments and to consider sending vaccination teams to key border crossings. Acknowledging the structural issues that underpin mistrust in the health sector, the CHWs also recommended the provision of stipends for volunteer health workers to do outreach and tackling the problem of frequent drugs stock outs, as part of bigger efforts to improve community members’ health-seeking experiences. The second set of recommendations were more directly targeted at short-term efforts to improve community engagement. These were piloted by the CHWs in January 2020 and then in February and March 2020 we conducted another round of research to consult communities on their effectiveness. In this paper we focus on two of these strategies as case studies.

Firstly, to address the issue of a lack of trust in the health system, based on past experiences and pre-emptive concerns, the CHWs proposed an “interface meeting” between community members and health staff from the PHUs, facilitated by the CHWs. Participants in these meetings were identified through the power mapping exercises conducted in the first stage of research and to represent community composition. The meetings started with the CHWs sharing their research findings, but their major aim was to facilitate a dialogue between community members and their PHU’s staff and to encourage both sides to express their concerns and be candid about their challenges. The CHWs emphasised that they wanted community members to have an opportunity to voice their anxieties around visiting the health centres and attending vaccination appointments, and to recount difficult experiences that had made them lose trust the health centres and staff. At the same time, they wanted to encourage the health staff to put their own challenges on the table, for example by explaining the daily realities of running a health clinic with limited resources and mostly staffed by volunteers. In the meetings, discussions also focused on the specific issue of parents being charged for vaccines and vaccination cards.

Interviews with both health staff and community members after the meetings suggested that they were found to be valuable on both sides, and respondents proposed that these kinds of meetings should be convened more frequently to be able to see both sides of the challenge and to find collective solutions. One respondent said:

“*[the most interesting part of the meeting for me] was when the health facility in-charge accepted all the criticisms and further explained that, not all the nurses in the centre are paid, and she told us she will have to hold a meeting with them*. *Well after sometime, the numerous complains we were getting have not been coming up*….*so I am sure she talked to them…..That is great!”*

Health staff also reported that the meeting had encouraged them to visit their catchment communities and their perception that this was improving uptake during outreach:

“…*we agreed as health staff to hold a meeting every weekend concerning these issues and we tried it on the first weekend after the interface meeting*, *we paid a visit to [community 1 and 2] for outreach they received us well and brought their children for vaccination.*”

Whilst many of the structural issues raised in the meetings could not be addressed at the community or even District level, simply offering a forum to have frank and respectful conversations was a starting point for efforts to rebuilt trust.

The second strategy was focused on leveraging local concepts, knowledge and experience to reframe discussions around vaccination. In our analysis workshop, there was consensus that the mainstream approach that marginalises or ignores or dismisses the role of traditional medicine and community knowledge was counterproductive. Traditional healers or birth attendants, for example, were shown to be relatively trusted and the CHWs suggested engaging them informally to ask their advice on how they could improve community engagement strategies around vaccination. In addition, during the period of community engagement, they trialled the use of local concepts of protection to stimulate dialogue around the role of vaccines. CHWs used the concept of ‘*tarma’* (or adapted it to other terms that specific communities were more familiar with like *mabukor*), a local word to refer to processes of protecting oneself from witchcraft and its manifestations in “country sick”. These rituals are common in Kambia District and include for example using water prepared by a traditional healer, smoke or herbs to create a protection field around one’s body. CHWs used this notion to leverage existing notions of prevention to start a conversation about how vaccines can help prevent infectious diseases, or “hospital sicks”. During their observations, CHWs noted that discussing the question of protection using local terms like *tarma* stimulated interesting responses. In one village for example, a TBA pointed out that while adults might have to travel and pay to “worship their body”, this protection for children is supposed to be free. Similarly, a mother noted that vaccines had meant that some illnesses had been “lost”, “which is just similar to the medicine we the big people use to ‘embalm’ ourselves for witch-guns or snakes”. In other words, she noted that vaccines could protect against illnesses and make sure they were no longer seen within communities, just like traditional medicines could protect people from witchcraft attacks.

## Discussion

The lessons from this project can be divided into two categories. Firstly, substantive findings that contribute to more contextual understandings of the social dynamics of vaccine confidence and hesitancy, including an insight into local drivers of trust. This responds directly to calls for deepening qualitative evidence on these dynamics and to continue expanding the geographical focus to include experiences from the Global South, as noted at the start. Secondly, process findings about the role of CHW-led research in strengthening community engagement strategies for vaccination.

The CHWs’ immersive research in their communities highlighted the complex interplay of structural factors and public engagement with vaccination in Sierra Leone’s borderland communities. Their findings support existing literature’s emphasis on the role of trust whilst also showing that how trust is built and lost is context specific. They showed that understanding challenges around vaccine uptake required engaging with much broader concerns about the functioning of the rural health system, previous experiences of service delivery and individual interactions with healthcare workers, including outside of vaccination encounters. Experiences of marginalisation or even humiliation for inhabitants of remote rural communities during encounters with health workers led to lower levels of trust in the health system, which translated into avoidance of health services and scepticism in relation to vaccines. Mistrust was exacerbated by structural challenges that undermined the quality of service provision and eroded relations between communities and their healthcare workers. Instead, expressions of trust centred on meaningful social relations, defined by social proximity, reliability and respect. This was associated for example with traditional healers but also with individual healthcare workers who made tangible efforts to accommodate patients’ perspectives and needs. These were key considerations in making health-seeking decisions, including whether to vaccinate one’s children. A careful description of community characteristics highlighted the specificity of borderland livelihoods, and how these are rarely accounted for in the organisation of vaccination campaigns. Similarly, power mapping exercises questioned the effectiveness of standardised approaches to mobilising certain kinds of leadership without an in-depth assessment of power dynamics within each community. In the past this had meant that trusted informal authorities had been excluded from engagement efforts. Informal relations were key, but often missed by researchers and practitioners focusing on formal healthcare encounters as an entry-point from which to understand vaccination. Overall, the findings encouraged the team to situate the vaccine encounter in broader everyday health-seeking experiences, as well as historical and contemporary drivers of mistrust. This also required broadening the lens from individual attitudes such as ‘hesitancy’ to consider how the organisation of vaccine deployment might be redesigned to ensure trustworthiness, and to adapt to the diversity of livelihoods, knowledge(s) and experiences within and between communities where campaigns take place.

Implementing some of the short-term recommendations based on these findings into a new community engagement strategy highlighted the relatively simple but effective lessons that respectful, two-way dialogue is an essential step towards restoring trust. The interface meetings could not address most of the structural problems of health service delivery, yet creating a forum where both community members and health workers could express their concerns, anxieties and limitations helped to improve relationships. For community members, for example, an open discussion about the fact that health workers are often not paid was useful for understanding the challenges to delivering the kind of services they expected. Similarly, leveraging existing local knowledge, using concepts from Themne cosmology, or involving traditional healers in discussions about how to improve vaccination strategies, not only improved communication but also ensured that communities were shown respect for their knowledge and expertise.

Our second round of research showed that these targeted interventions based on our findings were well received in the five communities, however it will be necessary to conduct longer-term research, including during outbreaks and vaccination campaigns, and to supplement qualitative findings with quantitative analysis of vaccination uptake. Whilst increased uptake is an undeniably important goal, we argue that the most significant lessons from this project emerged from the process of directly involving CHWs as community focal points and public health officials in knowledge production through immersive, participatory research. The project was co-designed from the start, in direct response to District public health officials’ concerns that there was a mismatch between the realities in borderland communities and standardised approached to community engagement around vaccination. Through our community-led data collection, analysis and operationalisation processes we were able to devise activities that were directly responsive to the experiences and concerns of residents of the areas where engagement was to take place. The research process itself created spaces to stage the kind of dialogue that we found to be necessary to start rebuilding trust between communities and health workers.

Having district public health officials involved from the design to the implementation and analysis stage and involving CHWs as community researchers in the process of knowledge production meant they felt ownership over the findings and were more likely to take the initiative to implement recommendations and change their own practice. An immediate example of this, was the fact that the same approach of community-led ethnographic research was replicated from May 2020, whereby the trained CHWs conducted research on experiences of COVID-19 and fed back into a newly established social science pillar within the District’s response to the pandemic.

The CHWs’ positionality as community focal points and go-betweens with health facilities also provided a particularly insightful viewpoint. On the one hand, they had a kind of access and ‘insider perspective’, that external professional researchers could not gain. On the other hand, CHWs’ dual role as representatives of the community and the health system, also meant that they could act as intermediaries. This undoubtedly created tensions in their roles and in how they were perceived in their communities. At the same time, it also meant that they developed new ways of understanding their communities’ health challenges and started dislodging some entrenched beliefs about the District’s vaccination performance. New strategies tried to replace efforts based on assumptions of a lack of knowkedge to centre local experiences and questions of trust. In addition, spending more time in these five villages, and changing the quality of interactions, as CHWs went in with a different perspective and an open mind as researchers, influenced their relationships with and perspectives of those whose health and wellbeing they look after. However, this also raised difficult questions about the limits of this kind of work in addressing the much larger challenges and structural inequities that the research process brought to light.

## Limitations

The project was not without its challenges. The short time frame of the project meant that we cannot show a direct effect of the community-led research on increased vaccination uptake. Our analysis focuses instead on evaluating the process of developing and implementing a model of community-led research and translating findings into operational recommendations. Similarly, due to limited funding this project could only target five border communities and only trained a very small number of CHW-researchers.

In addition, because of the nature of the research the CHWs had to be literate, and, due to limited education opportunities for women in this region, the DHMT were only able to identify only one female CHW to participate. Literacy has more broadly been identified as a barrier to entry for those wishing to become CHWs [35]. In future developments of this approach, we propose to integrate more inclusive approaches, such as using voice notes instead, or in addition to, ethnographic diaries, and opening up the range of ‘community focal points’ who might lead the research process. Related to this, although this was not explicitly stated during the project, the fact that they were provided stipends as researchers, in contrast to meagre (and often delayed) compensations provided for their health worker roles, it is possible that this could cause distortionary incentives and diverted CHWs’ attentions from their healthcare responsibilities. In the short-term we aimed to prevent this by ensuring that ethnographic observations could overlap with their daily operations. In the long-term, we would suggest exploring possibilities for integrating this research training in CHWs’ standard training package, as well as addressing the problem of low retribution for their healthcare work.

It is important to note that the proposed approach requires extensive facilitation from professional social scientists in all aspects of delivery, including in situating findings in theoretical analysis. Much like the model of ‘citizen science’, this process does not replace social science expertise but broadens the base of collaboration for co-producing knowledge about the social dimensions of vaccination.

## Conclusion

This paper highlights the role of community-led, participatory social science research as a key component for understanding and addressing context-specific vaccination challenges. Our pilot study involved CHWs in collecting and analysing data and translating findings into operational recommendations. Their work highlighted key issues of trust and the interplay between vaccine supply and demand, making a case for sustained dialogue and holistic health system strengthening to boost vaccine confidence.

There is scope for broader application of this community-led ethnographic approach in the field of vaccination, as well as other domains of global public health. This will help public health experts to identify important barriers and facilitators and devise more tailored and context-specific community engagement strategies that leverage existing community knowledge and directly respond to their concerns and lived experience. Even more importantly, the process of involving communities and low cadre service providers in the generation of knowledge and solutions, can help to strengthen relationships and to sustain meaningful dialogue to bolster vaccine confidence.

## Supporting information

S1 File Topic guides for interviews and FGDs. (DOCX)
