# Supplementary material for: Bringing the social into vaccination research: Community-led ethnography and trust-building in immunization programs in Sierra Leone
Source: PLoS One. 2021 Oct 22;16(10):e0258252. doi: 10.1371/journal.pone.0258252 (PMC8535180; doi:10.1371/journal.pone.0258252)
Supplement: S1 File — (DOCX) [file pone.0258252.s001.docx]

**S1 File. Topic Guides for Interviews and FGDs**

N.B. These are the broad lines of our project’s topic guides, they are semi-structured and served as pointers for more specific lines of questioning. These guides were then refined, including adding more specific directions for the purposes of our project, and they were made more specific to each community’s context through an iterative process of research and consultation.

1. **Topics for Interviews**

*Background Question*

1. Please tell me a little bit about yourself
2. What is your position in this community?
3. How long have you been in this community?

*Healthcare facilities & health-seeking*

1. What are the most common illnesses in this community? What can cause them?
2. How do you see the health system?
3. Where do you go if you or someone in your family is not well? Why?
4. What are the main/ closest healthcare facilities in this community?
5. What is the role of traditional healers in this community? How do people decide whether to go to the herbalist or elsewhere?
6. What is your experience of visiting a health facility (e.g. PHU or Government Hospital)? Can you give me an example?
7. What are some challenges you face in seeking healthcare in your community? Can you give me an example?
8. Which groups in your communities face more/less challenges in receiving healthcare?
9. Has this community experienced any disease outbreak (e.g. measles, Ebola, cholera etc?)
10. Can you remember what happened during that time? (encourage them to give examples and stories)
11. Do you think the Ebola outbreak had any impact in this community? If yes, can you give some examples?
12. If any other outbreak were to happen, do you think everything is in place to prevent it from having a negative impact? If yes, why? If not, why not? What could be improved

*Vaccination*

1. Please tell me about experiences of vaccination in this community?
2. When do people take vaccines? Where do they go?
3. How do people feel about routine vaccination?
4. How do people feel about campaigns (for example the recent measles vaccine campaign)
5. Do you think people in this community mostly agree to take vaccines (and why) or mostly disagree (and why)?
6. Why do you think these challenges occur?
7. What do you think works well during campaigns or routine vaccination?
8. What do you think does not work well?
9. How could the DHMT improve vaccination coverage?
10. How could the community leaders improve vaccination coverage?
11. How could CHWs improve vaccination coverage?

*Evaluating Community Engagement (for Phase II of research)*

Last month, we tried to implement some new strategies to talk with people in your community about vaccination and to address some of the concerns that they have around vaccines and healthcare more generally. These strategies were based on all the time we spent in your community listening to everybody here. We now want to ask your opinion on the community engagement activities we did and for you to help us improve this even more.

1. Did you attend the community interface meeting that we organised with the PHU staff?

- If yes:
  - Can you tell me what happened during the meeting?
  - What did you find interesting?
  - What did you think worked well?
  - What was not so successful?
  - What else do you think we should have discussed?
- If no: did you hear about it? What did you hear? What do you think we should have discussed?

1. Do you see any changes in the community or the PHU since the interface meeting took place?

- If yes: what kinds of changes? Why do you think these changes happened?
- If no: why not? Why do you think it is difficult for changes to happen?

1. What do you think needs to happen to improve these kinds of interface meetings?
2. I will now explain to you some of the other key strategies and messages we used and I would like you to tell me **for each one**:

- Do you think it is a good idea?
- What challenges might there be for implementing this strategy?
- How do you think it could be improved?

1. **Topics for FGDs**
2. *Health service providers: experiences & trust*

- What health services are available in this community?
- What do people do if they become sick?
- How would they describe experiences with doctors, nurses and health facilities? What about other services (e.g. traditional healer/ peppeh doctor/ TBAs etc)
- If somebody becomes sick, who would they trust the most to help? Who would you trust the least?

1. *Epidemics*

- What is this community’s recent experience with diseases like Ebola/ measles?
- What do you think can be done to prevent the spreading of those diseases in the future?

1. *Vaccination*

- What is the role of vaccination?
- What are some barriers to vaccination in this area?
- What are some rumours about vaccination in this area? Why do people have these ideas?
- What can improve vaccination access in this area?
- What is your experience of social mobilisation campaigns (e.g. for measles vaccination)?
- How would you improve those campaigns?

1. *Power Mapping*

- Identifying determinants of power (e.g. formal position, wealth, knowledge etc)
- Discussing the relationship between power and trust
- Identifying key individuals and describing the nature of their power in the community in relation to vaccination challenges and whether they are trusted
- Drawing relations between key individuals

1. *Rumour Tracking*

- Identifying key rumours: where do they spread? Why do people share them?
- Why might people hold these views?
- Discussing frequency and significance

1. **Topics for Participant Observation**

- Life in the community (socio-economic activities, history of the community, cross-border relations)
- Community authority structures
- Mapping of health services (from PHUs to traditional healers)
- Barriers to access to health services
- Trust in health services
- Experience of recent outbreaks (Ebola/ measles)
- Experience of vaccination and vaccination campaigns
- Barriers/ opportunities for vaccination
- Perspectives on social mobilisation around vaccination and suggestions for improvement
